# Improved Seam-Line Searching Algorithm for UAV Image Mosaic with Optical Flow

**DOI:** 10.3390/s18041214

**Published:** 2018-04-16

**Authors:** Weilong Zhang, Bingxuan Guo, Ming Li, Xuan Liao, Wenzhuo Li

**Affiliations:** 1 State Key Laboratory of Information Engineering in Surveying Mapping and Remote Sensing, Wuhan University, Wuhan 430079, China; zhangweilong@whu.edu.cn (W.Z.); liaoxuan@whu.edu.cn (X.L.); alvinlee@whu.edu.cn (W.L.); 2 Collaborative Innovation Center of Geospatial Technology, Wuhan University, Wuhan 430079, China; 3 School of Resource and Environmental Science, Wuhan University, Wuhan 430079, China

**Keywords:** UAV image, dynamic programming, seam-line, optical flow, image mosaic

## Abstract

Ghosting and seams are two major challenges in creating unmanned aerial vehicle (UAV) image mosaic. In response to these problems, this paper proposes an improved method for UAV image seam-line searching. First, an image matching algorithm is used to extract and match the features of adjacent images, so that they can be transformed into the same coordinate system. Then, the gray scale difference, the gradient minimum, and the optical flow value of pixels in adjacent image overlapped area in a neighborhood are calculated, which can be applied to creating an energy function for seam-line searching. Based on that, an improved dynamic programming algorithm is proposed to search the optimal seam-lines to complete the UAV image mosaic. This algorithm adopts a more adaptive energy aggregation and traversal strategy, which can find a more ideal splicing path for adjacent UAV images and avoid the ground objects better. The experimental results show that the proposed method can effectively solve the problems of ghosting and seams in the panoramic UAV images.

## 1. Introduction

Image mosaics have a long history starting in the early days of computer vision and photogrammetry. With the rise of UAV remote sensing technologies, this research has become paramount to many applications based on UAV survey including 3D reconstruction, ecological farming, disaster emergency management, and photography activity. These are due to UAV remote sensing technology’s strengths of low-cost, high–Speed, and easy accessibility [1,2,3,4,5]. However, there are three disadvantages, its low flight altitude, the camera perspective constraints, and the small coverage area of a single UAV image. In many applications mentioned above, in order to expand the image coverage area to capture more information from the target area, multiple UAV images are collected, leading to the need of mosaic multiple images to form a panoramic image. Furthermore, high-altitude wind has a significant impact on the UAV platform due to its light-weight, problems such as irregular image overlaps and uneven image exposure are introduced into the adjacent images [6]. Therefore, images captured from an unstable UAV platform will lead to a vulnerable stitched image with ghosting, blur, dislocation, and color inconsistency. Overall, there are many challenges about the state-of-the-art image mosaic methods.

In response to these difficulties, this paper proposes a new UAV image mosaic method. The method solves the dislocation and ghosting problem cause by selecting the optimal seam-line in the building-intensive areas. In this new method, we first introduce the optical flow to construct the energy function for seam-line searching, it can factor the image structure information into the seam-line optimization better. Secondly, a new seam-line search strategy is presented. In this method, its basic idea is to determine the geometric errors introduced by perspective errors, camera distortions, and radiation errors by analyzing the mapping relationships between the left and right images, then using these errors to aid in the seam-line search process.

## 2. Related Work

There are various methods for seamless mosaic of UAV remote sensing images have been investigated [7,8,9,10,11,12,13,14,15,16,17]. Among them, seam-line based methods are intended to reduce grayscale and geometric differences. They look for the least-cost seam-line in the overlapping region of adjacent images by constructing an energy function. This paper will focus on the seam-line search methods based on dynamic programming and optical flow.

### 2.1. Methods Based on Dynamic Programming

This is a kind of mainstream image mosaic method. The methods in [11,12,13] focus on the energy difference between the images and their effects are superior, but they still present some problems. For example, dynamic programming-based methods in [11,12,13] adopt Dijkstra’s shortest path algorithm to search for the optimal seam-lines, which address the ghosting and dislocation problems because of the movements of the objects and registration errors, but they suffer from low search efficiency and complex weight determination. The ant colony method in [14] is also based on dynamic programming, which can evade the areas where the color contrast is larger between images, while it will easily lead the search processing to the local optimum due to its sensitivity to the number of ants. Furthermore, there are some other methods based on the snake model [15], and some based on a morphological model [16,17]. Although these methods can almost ensure the consistence of the geometric structure and evade the phenomenon of ghosting in the overlapping regions under some conditions, they are still unable to ensure that ghosting and seams can be overcame at the same time—especially when there is a significant brightness difference between adjacent images. Meanwhile, these methods are unable to achieve satisfactory results when there are rich texture structures, registration errors, and radiation brightness differences between images. Furthermore, most of the current seam-line search methods based on dynamic programming theory rely strongly on image direction that leads to a low robustness with their energy functions.

### 2.2. Methods Based on Optical Flow

Optical flow is the pattern of apparent motion of objects, surfaces, and edges in a visual scene caused by the relative motion between an observer and a scene [18,19]. The American psychologist Gibson introduced the concept of optical flow in the 1940s [20]. Sequences of ordered images allow the estimation of motion as either instantaneous image velocities or discrete image displacements [21]. David and Weiss introduce gradient-based optical flow [22]. John, David, and Steven provide a performance analysis of a number of optical flow techniques. It emphasizes the accuracy and density of measurements [23]. So far, there are many methods to calculate the optical flow, and these methods have great differences. There is still no systematic classification of the existing methods. Here, we divide the optical flow calculation methods into the following four categories: methods based on gradient, methods based on matching, methods based on frequency, and methods based on Bayesian. Among them, gradient-based methods are simple computation and effective, so they have been widely studied. Lucas–Kanade method and Horn–Schunk method are representative methods, they are used to calculate the motion of a partial pixels of images (called sparse optical flow) and the motion of all pixels of images (called dense optical flow), respectively [24,25]. The energy function constructed in this paper needs the optical flow value of each pixel in the overlapping area of adjacent images, so we use the dense optical flow method to calculate them. In the image mosaic, using the methods based on optical flow to estimate the camera’s motion parameters has the following advantages over the methods based on feature matching. It is unnecessary to extract image features. They are not sensitive to noise. Moreover, they can be applied to complex scenes and do dense optical flow calculation on the entire image without extrapolation of interpolation. Nevertheless, methods based on optical flow also have some weaknesses. Specifically, feature matching–based methods can be applied to the adjacent image with large difference and correct mark the corresponding points of adjacent images. However, methods based on optical flow assume that the change between images is continuous and the difference between adjacent images is very small, which greatly limits the application of these methods. For UAV image mosaic, the difference between adjacent images may be very large due to the fast flight and illumination changes of UAV. Therefore, it is difficult to create UAV image mosaics only by the methods based on optical flow. Nonetheless, the optical flow information of pixels in the overlap area of adjacent images can well provide the structural information of the images, which is conducive to searching for the optimal seam-line [26].

In this paper, the optical flow information of the pixels in the overlapped area of the adjacent images is used to construct the improved dynamic programming energy function, trying to find the best seam-line between the adjacent images and realizing the seamless mosaic of UAV images. The reminder of this paper is organized as follows. In Section 2, we explain the methodology of our proposed new method based on Duplaquet’s method in detail. Experiments and results are described and analyzed in Section 3. Discussion and conclusions are drawn in Section 4.

## 3. Methodology

It is well–known that image registration is a key technology in the research of image mosaic method. The same is true of the research in this paper. Before the seam-line search, this paper uses the classic SIFT(scale-invariant feature transform)-based image feature extraction and matching algorithm for the registration of experimental images, in which the false matching points are removed by the RANSAC (random sample consensus) algorithm. Then, the experimental image pairs in this paper are transformed into the same coordinate system. Finally, these registered images are used for subsequent experiments.

### 3.1. Classic Duplaquet’s Method

In 1958, Bellman proposed the optimization theroy for multi-stage problems. He transformed the multi-stage process into a series of single-stage solution problems, and created a dynamic programming method [27]. Based on Bellman’s theory, Duplaquet proposed an improved method to search for image seam-lines based on dynamic programming idea [28]. Formula (1) is the energy criterion defined in the classic Duplaquet’s method

*C*(*x*, *y*) = *C_dif_* (*x*, *y*) − *λC_edge_* (*x*, *y*) (1)

where *C_dif_* (*x*, *y*) is the mean value of the gray scale difference of the pixel in the overlapping regions between adjacent images, *C_edge_* (*x*, *y*) is the gradient minimum of the pixel in the overlapping areas, *x*, *y* are the pixel coordinates, and λ is a weighting factor, which can be used to adjust the proportion of gray scale difference and structure difference in the energy function, the value of *λ* is −0.15 in classic Duplaquet’s method.

### 3.2. Problems Analysis of Duplaquet’s Method

**Figure 1:**
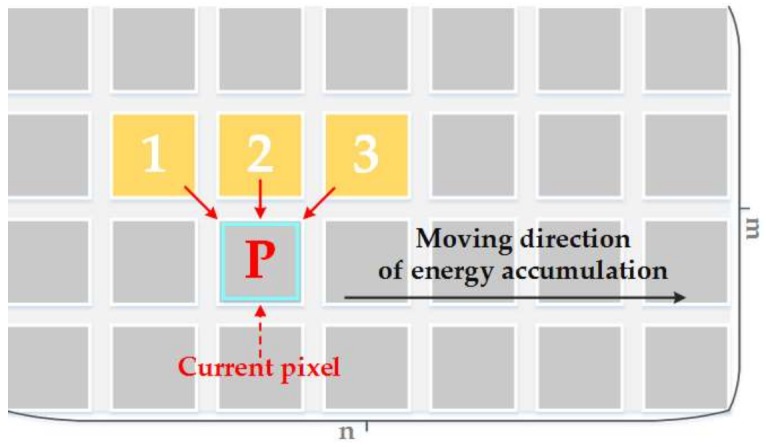
The schematic diagram of Duplaquet’s energy criterion.

**Figure 2:**
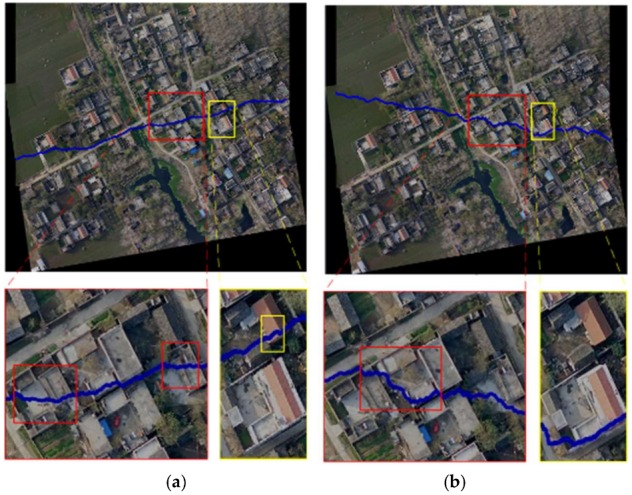
The mosaic results using the existing methods for two image pairs. (**a**) The Duplaquet’s method; (**b**) the method introduces the fourth horizontal direction based on the Duplaquet’s method.

The energy criterion in the Duplaquet’s method only considers the horizontal and vertical gradients, and compares the pixels in three adjacent directions near the current pixel, as shown in Figure 1. *P* is current pixel, *m* and *n* respectively present the pixel width and height of the overlapping region. When the overlapped region has dense tall ground objects (e.g., buildings or trees), the seam-lines output from the Duplaquet’s method are likely through the edges of the buildings due to the inconsistent deformation from the image point to the roof point (as in Figure 2). Thus, it is easy to produce ghosting and seams in the stitched images.

As shown in Figure 2. There are two experimental results based on the existing methods. The seam-lines across the houses can be easy to see in stitched images. Among them, the Figure 2a is the result of the Duplaquet’s method, the Figure 2b is the result of another existing method which introduces the fourth horizontal direction based on the Duplaquet’s method, its seam-line across the houses less than the Duplaquet’s method, but the seam-line still deviates from the ideal seam-line. Nowadays, some researchers believe that the energy function is poorly fitted, making it difficult to find the optimal seam-line. For this reason, these researchers attempt to modify the energy function based on dynamic programming. However, they overlook the optimality of the corresponding model. This also happens in the methods included in the OpenCV library. One of reasons for these problems is that the Duplaquet’s method cannot ensure the best seam-line by using the classical Sobel operator to calculate the approximate gradient of the pixels based on the horizontal and vertical templates (Formula (2)) without considering diagonal directions in the calculation process [29]. In Formula (2), *D_x_* and *D_y_* are the gradients of the pixel (*x*, *y*) in the vertical and horizontal directions, respectively.

(2) $$D_{x}=\left[\begin{array}{ccc} -1 & 0 & 1\\ -2 & 0 & 2\\ -1 & 0 & 1 \end{array}\right]\;\;D_{y}=\left[\begin{array}{ccc} -1 & -2 & -1\\ 0 & 0 & 0\\ 1 & 2 & 1 \end{array}\right]$$

Specifically, the Duplaquet’s method has the following three problems: (1) The gradient guidance direction of the energy function does not support omnidirectional searching. (2) The energy function is direction-dependent, and the energy aggregation considers only three directions, and the direction of energy traversal are limited from left to right, as well as from top to bottom. (3) The energy function getting local optimal solution is easy due to the impact of the two factors mentioned above. These will directly lead to the optimal seam-line susceptible to dense ground objects.

### 3.3. Improved Seam-Line Search Method

This paper introduces the optical flow value of the pixels in the overlapped regions for seam-line searching, and proposes a new method for finding optimal seam-lines by improving gradient guidance direction, energy accumulation directions that include energy aggregation directions, and energy traversal direction.

#### 3.3.1. Gradient Calculation

The Duplaquet’s method only considers the horizontal and vertical gradients in the energy criterion; it often fails to obtain the optimal seam-line. To solve this problem, this paper uses a new gradient operator based on the classical Sobel operator, which considers eight-directional neighborhood information of current pixel and the similarity of its surrounding structure [30]. The new approach of gradient calculation is

(3) $$D_{0^{{}^{\circ}}}=\left[\begin{array}{ccc} 1 & 2 & 1\\ 0 & 0 & 0\\ -1 & -2 & -1 \end{array}\right]\;D_{45^{{}^{\circ}}}=\left[\begin{array}{ccc} 2 & 1 & 0\\ 1 & 0 & -1\\ 0 & -1 & -2 \end{array}\right]\;D_{90^{{}^{\circ}}}=\left[\begin{array}{ccc} 1 & 0 & -1\\ 2 & 0 & -2\\ 1 & 0 & -1 \end{array}\right]\;D_{135^{{}^{\circ}}}=\left[\begin{array}{ccc} 0 & -1 & -2\\ 1 & 0 & -1\\ 2 & 1 & 0 \end{array}\right]\;D_{180^{{}^{\circ}}}=\left[\begin{array}{ccc} -1 & -2 & -1\\ 0 & 0 & 0\\ 1 & 2 & 1 \end{array}\right]\;D_{225^{{}^{\circ}}}=\left[\begin{array}{ccc} -2 & -1 & 0\\ -1 & 0 & 1\\ 0 & 1 & 2 \end{array}\right]\;D_{270^{{}^{\circ}}}=\left[\begin{array}{ccc} -1 & 0 & 1\\ -2 & 0 & 2\\ -1 & 0 & 1 \end{array}\right]\;D_{315^{{}^{\circ}}}=\left[\begin{array}{ccc} 0 & 1 & 2\\ -1 & 0 & 1\\ -2 & -1 & 0 \end{array}\right]$$

In Formula (3), *D***_0°_**, *D***_45°_**, *D***_90°_**, *D***_135°_**, *D***_180°_**, *D***_225°_**, *D***_270°_**, *D***_315°_** are the gradients of pixel (*x*, *y*) in eight directions, respectively.

#### 3.3.2. Directionality of Energy Accumulation

**Figure 3:**
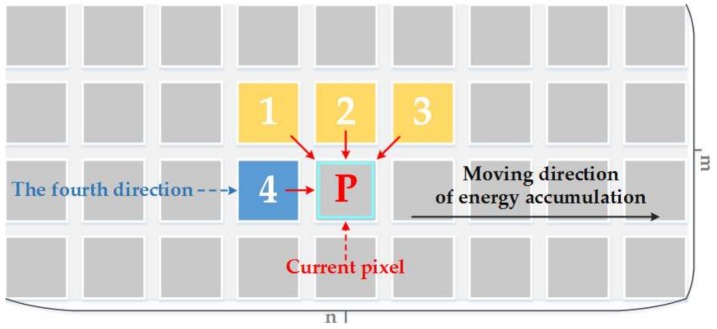
A schematic diagram of energy accumulation by improving energy guidelines.

In order to solve the direction-dependent problem in energy accumulation, this paper introduces a fourth horizontal direction in energy accumulation, as is shown in Figure 3. This change can get better seam-line which can be seen easily in Figure 2b. It is closer to the ideal seam-line by using this method than the Dulapquet’s method, but it is obviously insufficient.

**Figure 4:**
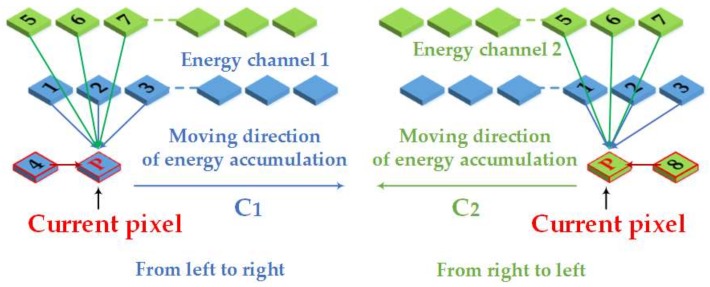
A schematic diagram of our search strategy.

Since the optimal seam-line searching is not only affected by the directions of energy aggregation, but also affected by the directions of energy traversal. Therefore, this paper redefines the new energy criterion and adds the new aggregate directions to our dynamic programming algorithm with a stereo dual-channel energy accumulation strategy. It improves the searching scheme of optimal seam-line. As shown in Figure 4, there is a schematic diagram of our optimal seam-line search strategy, which optimizes the seam-line search criteria by detecting the eight pixels (contain the horizontal direction) surrounding the current pixel.

In Figure 4, *P* is the current pixel. This paper redefines the nine related directions surrounding *P* as follows: 0 (initial invalid direction), 1 (top-left of *P* for energy channel 1), 2 (top of *P* for energy channel 1), 3 (top-right of *P* for energy channel 1), 4 (left of *P* for energy channel 1), 5 (top-left of *P* for energy channel 2), 6 (top of *P* for energy channel 2), 7 (top-right of *P* for energy channel 2), 8 (right of *P* for energy channel 2). Seam-line searching is an aggregation process of minimum energy. Each seam-line consists of neighborhood pixels with smallest energy value. In our method, the longest seam-line is the optimal seam-line.

#### 3.3.3. Calculation of Optical Flow Value of Pixels in the Overlapped Region

In this paper, in order to take into account the image structure information better in the overlapped region of the adjacent images, we use the optical flow value of pixels in the overlapped region as a constraint condition for the construction of seam-line energy function. According to Section 2, this paper uses a dense optical flow method for optical flow calculation. The H–S method proposed by Horn and Schunck is a very popular dense optical flow method, which is easy to calculate [25]. The pixel displacement in the overlapping area can be calculated by H–S method, it can obtain the optical flow value of each pixel in the overlapped region. L–K is a sparse optical flow method, which can only calculate the optical flow value of part pixels. Others need to be obtained by interpilation. This is also the main reason for using the H–S method in this paper. Formula (4) is H–S method’s objective function.

(4) $$\underset{u,v}{\min}E_{flow}(u,v)=\iint \left[({\left(T(x,y)-I(x+u,y+v)\right)}^{2}+a\cdot (u_{x}^{2}+u_{y}^{2}+v_{x}^{2}+v_{y}^{2})\right]dxdy$$

In Formula (4), *E_flow_* is the value of optical flow, *u* and ***v*** is the displacement in the *x* and *y* axis directions. *T* is the reference image, *I* is the current image, *a* is a weight factor, *u_x_*, *v_x_*, *u_y_*, *v_y_* are the first derivative of *u* and *v* in the *x* and *y* directions, respectively.

#### 3.3.4. Energy Function

Based on the analysis of the theoretical model, we constructed a mathematical abstract expression of the theoretical model. Assuming that image ***f***_1_ and image ***f***_2_ are an original image pair to be stitched, the energy function is defined as

(5) $$\begin{array}{l} E=\sum_{(x,y)\in O}B(x,y)\sigma (|f_{1}(x,y)-f_{2}(x,y)|)+\sum_{(x,y)\in O}\left(\max\left(\underset{0\leq k\leq 7}{d_{k}}(f_{1}(x,y))-\underset{0\leq k\leq 7}{d_{k}}(f_{2}(x,y))\right)\right)\\ +\sum_{(x,y)\in O}E_{flow}(x,y)+\sum_{(x,y)\notin O}N(x,y) \end{array}$$

In Formula (5), *E* is the energy value of the current pixel, *B* (*x*, *y*) determines whether the current pixel *P* (*x*, *y*) is in the boundary of the overlapped region, when *B*(*x*, *y*) =1, it means that it is not in the boundary region, and when *B*(*x*, *y*) = 10, it is in the boundary region. σ(*) is the Gaussian smoothing term, which uses the information in the local window to enhance the local influence of the current pixel. *f*_1_(*x*, *y*), *f*_2_(*x*, *y*) are pending images to be stitched. *O* is the overlapped area. *d*(*) represents the gradient function of one of the eight directions. *N*(*x*, *y*) is the energy value of the invalid area, which is the constant term, and the value is 100 times larger than the maximum value of *O*. *E_flow_*(*x*, *y*) is the optical flow constraint item. In the actual processing, each data item and smoothing item are to be normalized, and the boundary effect is considered. That is, a large weight is given at the boundary area and the invalid area.

#### 3.3.5. Computation Procedure

**Figure 5:**
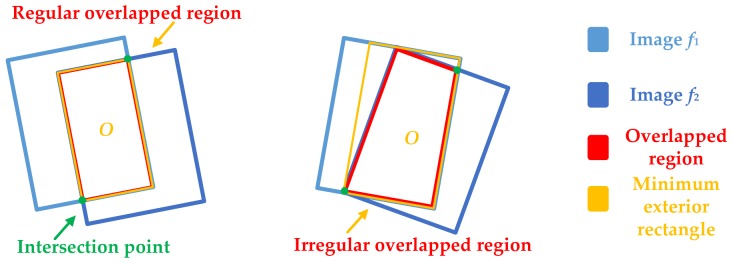
Processing principle of irregular overlapped region: (**a**) Regular overlapped region; (**b**) Irregular overlapped region.

Because the overlapped region of adjacent UAV images is often irregular, it needs to be handled properly to facilitate the calculation. As Figure 5 shows, the irregular overlapped area is in Figure 5b, it can be extended to a regular area by using the minimum exterior rectangle of the overlapped region. Let us say that the overlapped region is *m* × *n*.

**Figure 6:**
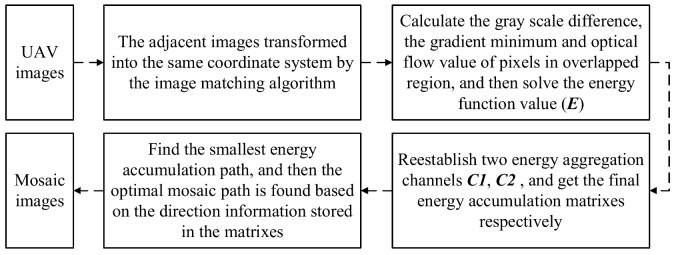
A flow chart of our method.

The image energy function *E* can be calculated by Formulas (3)–(5). A method flow chart of this paper is shown in Figure 6.

In the actual calculation process, each data item and smoothing item should be normalized. Furthermore, the boundary effect of overlapped regions needs to be taken into account, i.e., assigning a greater weight to invalid regions and boundaries. Therefore, the specific steps of our method are as following: Define the overlapped region of the adjacent images to be *O*, the buffer area of *O* boundary is *W* (set its width as 20 pixels, and *W* is an empirical value, the invalid area is *N* (extend area), and the boundary intersection *J*. Set *W* Є [1,10], the closer to the boundary, the larger the value is, and set *N* = 100×max (*O*), *J* = −1000×max (*O*)). At the same time, energy aggregation channels *C1* and *C2* have the same size as the minimum exterior rectangle; each pair of corresponding elements in these two matrices hold two scalar numbers representing the current aggregation value and the current path direction of the seam-line. For the first row of the matrices *C1* and *C2* assigned with the first row of *E* as the initial value, and set them corresponding direction as zero. The energy aggregation channel matrixes start to make a difference from the second row, which are divided into two aggregation processes from left to right and from right to left (see in Figure 4). For the energy aggregation channel *C1*, its aggregation process is from the left to the right; the current pixel only considers the directions of 1, 2, 3, 5, 6, 7, and 4. For the energy aggregation channel *C2*, its aggregation process is from the right to the left, and the current pixel only considers the directions of 1, 2, 3, 5, 6, 7, and 8. When the aggregation is finished, the minimum energy values are found from the last row in *C1* and *C2* respectively, and then an optimal mosaic path is found based on the direction information stored in the matrixes. In addition, in order to ensure that the seam-lines start and end at the intersection points, this paper selects two special intersection points (see that in Figure 5) that have the smallest energy value above, so that the seam-lines can be guided and adsorbed.

## 4. Experimental Results Analysis

### 4.1. Experimental Environment and Data

**Figure 7:**
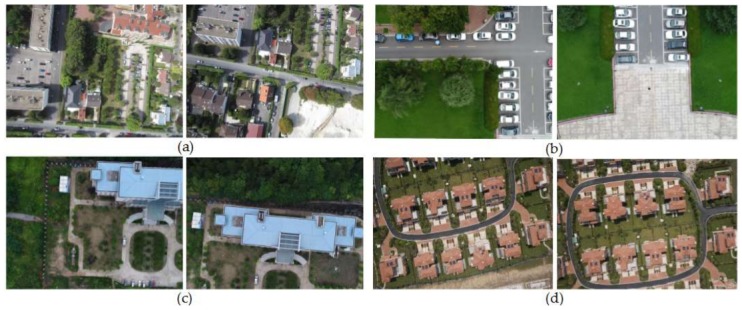
Four experimental UAV image pairs from four data sets: (**a**) The first image pair; (**b**) the second image pair; (**c**) the third image pair; (**d**) the fourth image pair.

In order to verify the effectiveness of our method (Our-flow-DP), this paper not only utilized the UAV images from different regions with different flight altitudes and cameras, but also compared the experimental results with the classic Duplaquet’s dynamic programming method using three search directions (Duplaquet3-DP), dynamic programming method based on Duplaquet using four search directions (Duplaquet4-DP), and the dynamic programming methods from OpenCV (OpenCV-DP). In this paper, we used Visual C++ based on OpenCV open source library to program the proposed improvement method. The experimental images are divided into four data sets; among them, the data in Figure 7a were acquired by Canon IXUS 220HS (Canon, Oita, Japan) in Paris, the height of the UAV is approximately 250 m. The data in Figure 7b were acquired by DJ-Phantom4 (DJ, Shenzhen, China) at Wuhan University square, the height of the UAV is approximately 115 m. The data in Figure 7c were acquired by DJ-Phantom4 (DJ, Shenzhen, China) at Hongshan district of Wuhan city, the height of the UAV is approximately 116 m. The data in Figure 7d were acquired by ILCE-QX1 (Sony, Chonburi, Thailand) in Jiashan County, China, the height of the UAV is approximately 150 m. The experimental computer environment is Windows 7 operating system, CPU: Intel (R) Core (TM) i7-4790, RAM: 32 GB.

### 4.2. Experimental Results Analysis

#### 4.2.1. Comparison of Mosaic Results with Four Different Image Pairs

**Figure 8:**
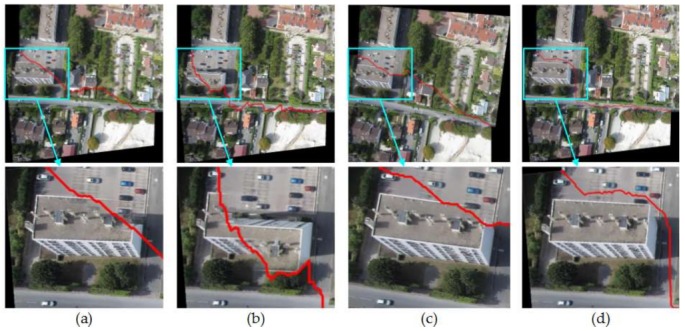
The seam-lines of different search methods for Figure 7a: (**a**) Dulapquet3-DP; (**b**) Dulapquet4-DP; (**c**) OpenCV-DP; (**d**) Our-Flow-DP.

**Figure 9:**
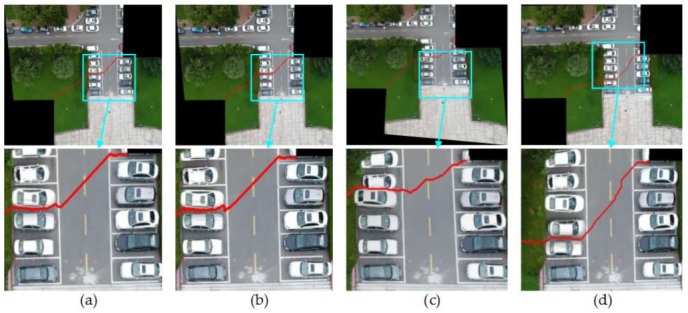
The seam-lines of different search methods for Figure 7b: (**a**) Dulapquet3-DP; (**b**) Dulapquet4-DP; (**c**) OpenCV-DP; (**d**) Our-Flow-DP.

**Figure 10:**
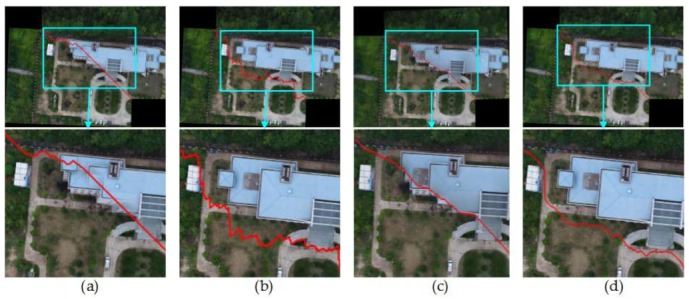
The seam-lines of different search methods for Figure 7c: (**a**) Dulapquet3-DP; (**b**) Dulapquet4-DP; (**c**) OpenCV-DP; (**d**) Our-Flow-DP.

**Figure 11:**
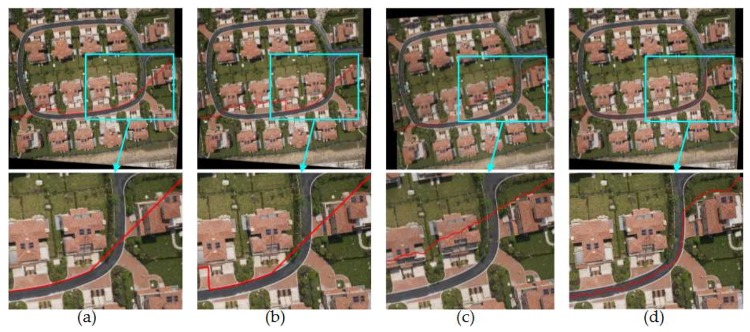
The seam-lines of different search methods for Figure 7d: (**a**) Dulapquet3-DP; (**b**) Dulapquet4-DP; (**c**) OpenCV-DP; (**d**) Our-Flow-DP.

So as to verify the effect of this paper proposed method, Duplaquet3-DP, Duplaquet4-DP, OpenCV-DP, and Our-Flow-DP were used to search the optimal seam-lines of image pairs in Figure 7 with irregular overlapped regions. Figure 8, Figure 9, Figure 10 and Figure 11 are the respective results. It can be seen from Figure 8, Figure 9, Figure 10 and Figure 11 that the optimal seam-lines are obviously different with the four test methods. From the local zoom view of Figure 8, Figure 9, Figure 10 and Figure 11, we can find that the optimal seam-lines searched by Our-Flow-DP are basically following along the road direction, which avoid the ground buildings, this will greatly reduce the probability of dislocation and ghosting because of image geometric errors. The other three methods place the seam-lines across the edges of houses, and present a ghosting and seam phenomenon. Especially in Figure 8, the other three methods have poor mosaic effects due to the dense distribution of buildings and the large changes in height. Furthermore, there was a problem of house information loss around seam-line edge in stitched images.

#### 4.2.2. Comparison of Four Methods under the Condition of Image Rotation

**Figure 12:**
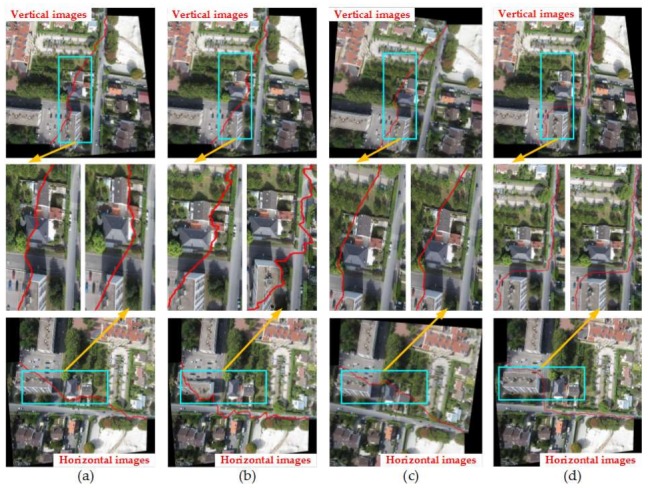
The seam-lines of four search methods for Figure 7a: (**a**) Dulapquet3-DP; (**b**) Dulapquet4-DP; (**c**) OpenCV-DP; (**d**) Our-Flow-DP.

**Figure 13:**
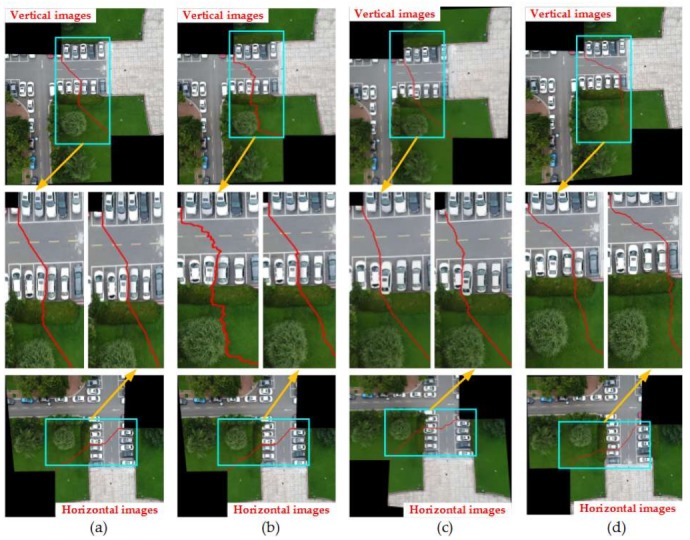
The seam-lines of four search methods for Figure 7b: (**a**) Dulapquet3-DP; (**b**) Dulapquet4-DP; (**c**) OpenCV-DP; (**d**) Our-Flow-DP.

**Figure 14:**
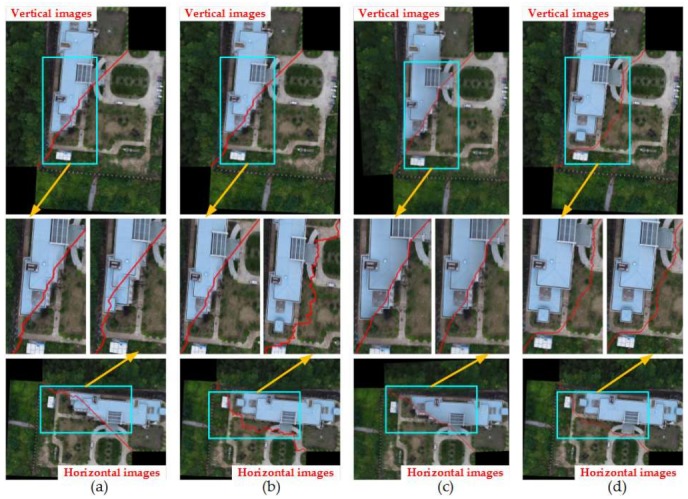
The seam-lines of four search methods for Figure 7c: (**a**) Dulapquet3-DP; (**b**) Dulapquet4-DP; (**c**) OpenCV-DP; (**d**) Our-Flow-DP.

**Figure 15:**
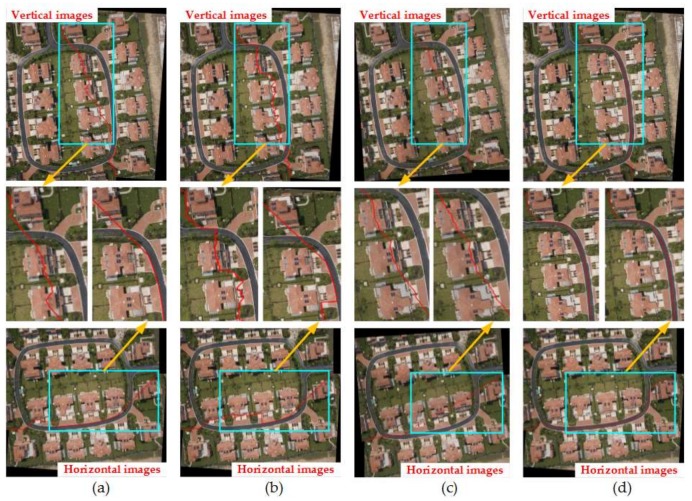
The seam-lines of four search methods under different situations for Figure 7d: (**a**) Dulapquet3-DP; (**b**) Dulapquet4-DP; (**c**) OpenCV-DP; (**d**) Our-Flow-DP.

Images in Figure 7 were rotated from the horizontal to the vertical firstly. Then, we used the four methods mentioned above to search the optimal seam-lines for vertical and horizontal images respectively. Figure 12, Figure 13, Figure 14 and Figure 15 show the results of them. In Figure 12, Figure 14, and Figure 15, the partially enlarged pictures illustrated that the optimal seam-lines searched by Our-Flow-DP basically no change before and after rotation, they always were good at avoiding the ground buildings and tall trees in the overlapped regions of adjacent images. In Figure 13, our seam-lines changed slightly, but they were less affected by the cars and tall trees in the overlapped regions than others, and the directions and movements of the seam-lines basically avoided the cars. In contrast, the seam-lines of the other three methods all crossed the edges of the buildings in different places before and after rotation, and the directions and movements of the seam-lines have an obvious change in Figure 12, Figure 14, and Figure 15. In Figure 13, the seam-lines of Duplaquet4-DP is more susceptible to tall trees than Duplaquet3-DP and OpenCV-DP. From the above results analysis, Our-Flow-DP is more independent than the other three methods in direction, and it can best avoid houses and tall trees for the best seam-lines searching when there are a large number of buildings and tall tress distribution in images, this is crucial for finding the most suitable seam-lines for adjacent images. Therefore, due to the specific improvements to the above issues of dynamic programming mentioned in Section 3.3, our method has advantages in adaptability and robustness for different UAV images. The minimum value of our energy function is almost no relationship to the direction of energy aggregation and traversal, and it can better take into account the structural information of the adjacent images.

#### 4.2.3. Efficiency Comparison of Our Energy Accumulation Processing

The improved method proposed in this paper found the almost best seam-lines in the previous experiments. Since Our-Flow-DP is based on the classical Duplaquet method, this section will compare the energy accumulation time efficiency of Duplaquet3-DP, Duplaquet4-DP, and Our-Flow-DP. Firstly, we assumed that Our-Flow-DP, Duplaquet3-DP, and Duplaquet4-DP could find the same optimal seam-lines. Their time efficiency difference can be quantitatively analyzed from the method’s complexity. In this paper, the direction of energy aggregation form three aggregation directions increased to eight is mainly an improvement. Setting that the time complexity of the Dulapquet3-DP is *O*(*z*^3^), the time complexity of the Dulapquet4-DP is *O*(*z*^4^), and Our-Flow-DP is *O*(*z*^8^), where *z* is the total number of pixels within the minimum exterior rectangle of the overlapped region, *z* can be expressed as the product of *m* and *n*, *m* is the width of the minimum exterior rectangle and *n* is the length of the minimum exterior rectangle. Both *m* and *n* are measured by unit pixel. However, because the local energy minima exists in the energy function of the Duplaquet3-DP and Duplaquet4-DP, they result in a lot of time consumption. Therefore, the above assumption is invalid, that is to say, they cannot get the same optimal seam-lines.

Four experimental image pairs were selected in Figure 7 to verify the above conclusions. In order to speed up the calculation, it is generally necessary to zoom the image at a certain scale. Therefore, the size of the overlapped region is not same to the size of the original image overlapped region. The experimental results can be seen in Table 1. The efficiency of our method is more than 37–148 times that of the other three methods. It proved the convergence speed of our energy function was faster than others. In addition, it further pointed out that the theory and the results of the proposed method were obviously different with the classic Duplaquet’s method. The theoretical improvement and experimental comparison have proven that this paper proposed a global and non-direction optimization method, which not only has the best seam-line, but also has better time efficiency.

## 5. Conclusions

This paper selected the essential problems of dynamic programming algorithms for image seam-line optimization, and introduced the optical flow value of the pixels in the overlapped regions for seam-line searching. At last, on the basis of classic dynamic programming algorithm, this paper proposes a new improved dynamic programming algorithm to search the optimal seam lines. Meanwhile, this paper carried out a detailed theoretical study and a lot of UAV image mosaic experiments. The superiority and efficiency of the method proposed in this paper are verified by the credible experiments of different image pairs with irregular overlapped regions. It can be seen from the experimental results in this paper, the UAV image mosaic results are better than the comparison methods. Furthermore, the proposed method is proven invariant to image rotation, and the improved dynamic programming algorithm works more efficiently. It is worth mentioning that the improved method in this paper is even better than the OpenCV method, which is an open source method and has been constantly updated. In the future, we will continue to improve our existing deficiencies to achieve a more perfect and robust method of rapid UAV image mosaic. In addition, a real-time UAV image mosaic will be our main research direction.

## Figures and Tables

**Table 1:** Time efficiency comparison of energy accumulation processing with different methods

Image Pair	Figure 7a		Figure 7b		Figure 7c		Figure 7d	
Rotation	Horizontal	Vertical	Horizontal	Vertical	Horizontal	Vertical	Horizontal	Vertical
z = m × n	956 × 522		635 × 362		745 × 560		1473 × 642	
Location	Paris		Square		Hongshan		Jiashan	
Duplaquet3-DP	5088 ms	4911 ms	1625 ms	1538 ms	3373 ms	3244 ms	14,501 ms	14,503 ms
Duplaquet4-DP	5075 ms	4862 ms	1580 ms	1516 ms	3321 ms	3238 ms	14,547 ms	14,229 ms
Our-Flow-DP	90 ms	55 ms	43 ms	24 ms	76 ms	44 ms	201 ms	98 ms
Multiple	56	89	38	64	44	74	72	148
	56	88	37	63	43	73	72	145
